# Prevalence and knowledge about acute mountain sickness in the Western Alps

**DOI:** 10.1371/journal.pone.0291060

**Published:** 2023-09-14

**Authors:** Marc Moritz Berger, Anika Hüsing, Nicolai Niessen, Lisa Maria Schiefer, Michael Schneider, Peter Bärtsch, Karl-Heinz Jöckel

**Affiliations:** 1 Department of Anesthesiology and Intensive Care Medicine, University Hospital Essen, University Duisburg-Essen, Essen, Germany; 2 Institute of Medical Informatics, Biometry and Epidemiology, University Hospital Essen, Essen, Germany; 3 Department of Internal Medicine, Klinikum Garmisch-Partenkirchen, Garmisch-Partenkirchen, Germany; 4 Paracelsus Medical University, Salzburg, Austria; 5 Institute for Health Services Research and Clinical Epidemiology, Philipps-Universitaet Marburg, Marburg, Germany; 6 Department of Internal Medicine, University Hospital Heidelberg, Heidelberg, Germany; University of Ljubljana, SLOVENIA

## Abstract

**Objective:**

To assess the prevalence of acute mountain sickness (AMS) in 1370 mountaineers at four different altitudes in the Western Alps. We also examined the influence of potential risk factors and the knowledge about AMS on its prevalence.

**Methods:**

In this observational cross-sectional study AMS was assessed on the day of ascent by the Lake Louise score (LLS, cut-off ≥3, version 2018) and the AMS-Cerebral (AMS-C) score of the environmental symptom questionnaire (cut-off ≥0,70). The latter was also obtained in the next morning. Knowledge regarding AMS and high-altitude cerebral edema (HACE) and the potential risk factors for AMS were evaluated by questionnaires.

**Results:**

On the day of ascent, the prevalence of AMS assessed by the LLS and AMS-C score was 5.8 and 3.9% at 2850 m, 2.1 and 3.1% at 3050 m, 14.8 and 10.1% at 3650 m, and 21.9 and 15% at 4559 m, respectively. The AMS prevalence increased overnight from 10.1 to 14.5% and from 15 to 25.2% at 3650 m and 4559 m, respectively, and was unchanged at 2850 m and 3050 m. A history of AMS, higher altitude, lower degree of pre-acclimatization, and younger age were identified as risk factors for developing AMS. Slow ascent was weakly associated with AMS prevalence, and sex and knowledge about AMS and HACE were indistinct.

**Conclusion:**

AMS is common at altitudes ≥ 3650 m and better knowledge about AMS and HACE was not associated with less AMS in mountaineers with on average little knowledge.

## 1. Introduction

Ascent to altitudes above 2000–2500 m bears the risk of developing acute mountain sickness (AMS), the most frequent acute high-altitude illness [1]. Headache is the cardinal symptom of AMS and is usually accompanied by additional symptoms such as anorexia, nausea, dizziness, malaise, sleep disturbance or a combination thereof [2]. AMS may progress to life-threatening high altitude cerebral edema (HACE) if not treated adequately.

The most frequent diagnostic tools [3] are the Acute Mountain Sickness-Cerebral (AMS-C) score of the environmental symptom questionnaire [4] and the Lake Louise score (LLS) [5]. In 2018, the LLS, has been revised [2] and sleep disturbance was removed from the diagnostic criteria because disturbed sleep at altitude is not closely related to AMS. In the present study, the LLS and the AMS-C score were obtained at four different locations in the Western Alps, at which AMS had also been assessed about 30 years ago [6]. The major objective of this study was to assess the prevalence of AMS, to identify the associated risk factors, and to investigate whether better knowledge regarding AMS and HACE is associated with a lower AMS prevalence and severity. A secondary goal was identifying changes in the frequency of AMS symptoms over 30 years at these 4 locations.

## 2. Methods

### 2.1 Study design and setting

This observational cross-sectional study was performed in the Western Alps at the Konkordia hut (2850 m; between 3.9.–16.9.2018), Finsteraarhorn hut (3050 m; between 7.7.–15.7.2018), Mönchsjoch hut (3650 m; between 17.6.–30.6.2018), and Margherita hut (4559 m; between 22.7.–1.8.2018). All mountaineers arriving at the huts were invited to participate in the survey by personal communication and by information posters placed at the respective hut entrance. The study was granted an exemption by the Ethical Committee Salzburg, Austria, as the survey did not include any identifying data. The requirement for obtaining informed consent was waived. None of the authors had access to information that could identify individual participants during or after data collection. Data from 29 underage participants were excluded from the analyses.

### 2.2 Study protocol

Within 6 hours after arrival at the hut and without assistance from the investigators the participants completed a questionnaire that was available in English, German, Italian, and French. The questionnaire consisted of two parts, a general part to be filled out once after arrival at the hut, and questionnaires assessing symptoms of AMS. In those who stayed at the hut overnight, assessment of AMS was repeated in the morning of the next day.

### 2.3 Questionnaires

The first part consisted of 5 questions evaluating the knowledge about acute high altitude illnesses (S1 Questionnaire) and of 28 questions addressing demographic data, altitude experience, the current ascent and health related information, and whether professional mountain guides or tour guides certified by national alpine clubs were engaged. For clarification, non-professional tour guides underwent guide-training organized by alpine clubs for experienced members and are then allowed to lead groups of other club members. However, they are less experienced in guiding than professional mountain guides. The second part of the questionnaire assessed the prevalence and severity of AMS.

### 2.4 Assessment of AMS

AMS was assessed with the AMS-C score and the LLS on the day of arrival and by the AMS-C score only on the next morning.

The AMS-C score of the abbreviated validated version of the ESQ consists of 11 items, graded from 0 (no symptoms) to 5 (very strong symptoms) [7]. Calculation of the score is based on factorial weights of each item, for details see [4]. An AMS-C score ≥ 0.7 indicates AMS.

The LLS was calculated according to the 2018 revised version [2], which is composed of the following symptoms graded from 0 (no symptoms) to 3 (severe symptoms): headache, gastrointestinal symptoms, fatigue and/or weakness, dizziness/light-headedness. A LLS of ≥ 3 in presence of headache and at least one other symptom indicates AMS.

Of note, several studies have shown that an AMS-C cut-off score of 0.70 corresponds to a LLS of about 5 [3, 8]. Thus, those with AMS fulfilling the criterion by the AMS-C score have somewhat more severe AMS than those with LLS > 3.

### 2.5 History of AMS

Study participants were asked how often (never = 0 points; rare = 1 point; often = 2 points; regularly = 3 points) typical symptoms of AMS (headache, gastrointestinal symptoms (i.e., loss of appetite, nausea, vomiting), dizziness, insomnia, and peripheral edema) occurred during previous sojourns at altitudes > 3000 m. Individuals that never or seldom had headache (≤ 1 point) at high altitude and who had a total score < 4 were considered not susceptible to AMS [9]. When exposures above 3000 m were < 1 day per year, participants were grouped into a separate category.

### 2.6 Pre-acclimatization and speed of ascent

Pre-exposure was assessed by calculating the days spent > 3000 m during the previous 2 months, not including the present climb [9]. Those mountaineers having spent at least 5 days above 3000 m during the preceding 2 months were considered to be well acclimatized. The speed of ascent was expressed as the number of days spent above 2000 m until the study location was reached, including the arrival day [9]. Slow ascent was defined as an ascent of ≥ 2 days to 2850 m and 3050 m, ≥ 3 days to 3650 m, and ≥ 4 days to 4559 m.

### 2.7 Knowledge about AMS and HACE

The questions and the score derived from the answers are described in S1 Questionnaire. All items of the questionnaire are considered to address essential knowledge for lay people traveling to high altitude by international experts as described by Berendsen et al. [10]. The maximum attainable knowledge score was 12 points.

### 2.8 Comparison of AMS symptoms with historic data

Since the AMS-C score or the LLS were not used in 1990 [6], comparison was limited to the symptoms headache, gastrointestinal upset and dizziness, which were obtained and graded from 0 to 3 points in both studies.

### 2.9 Data analysis

The results of the questionnaires were entered into a database and all samples were double checked by two different investigators. Proportions of missing data were below 5% for most variables, therefore the available data were analysed without imputation. The data is described as assessed per hut, categorical variables are presented as count and percent, continuous and score data is presented as mean and standard deviation or as median and interquartile range for potentially skewed data. Logistic regression was used to investigate the association between AMS (binary, according to AMS-C score or LLS) and potential or established risk factors, thus allowing for covariate adjustment. Quantitative exposure data, e.g., for age or knowledge regarding AMS and HACE, was considered as linear exposure effect in logistic regression. Because fewer persons provided data on the morning score, and correspondingly data from the evening score from mountaineers staying overnight were reduced, we used these data only in sensitivity analyses to assess the stability of our estimated associations.

## 3. Results

### 3.1 Study population

The characteristics of the study population are shown in Table 1. In total, 1370 mountaineers (66% male, 33% female, 1% unknown) were studied. Of these, 738 (54%) had an overnight stay at one of the huts representing 96%, 100%, 92%, and 94% of the registered overnight stays at the Konkordia hut (2850 m), Finsteraarhorn hut (3050 m), Mönchsjoch hut (3650 m), and Margherita hut (4559 m), respectively. 632 (46%) mountaineers were day tourists, predominantly at the Mönchsjoch and Margherita hut. The percentage of day tourists included in the study is unknown, because day tourists are not registered in the huts.

**Table 1:** Characteristics of survey respondents, data presented as mean ± standard deviation [N missing], median [interquartile range], or count (%).

Characteristics	2850 m (Konkordia hut)	3050 m (Finsteraarhorn hut)	3650 m (Mönchsjoch hut)	4559 m (Margherita hut)
Participants (all)	212	98	629	431
• with overnight stay	202 (95%)	97 (99%)	179 (28%)	260 (60%)
• day tourists / early risers	10 (5%)	1 (1%)	450 (72%)	171 (40%)
Gender				
• female	62 (29%)	29 (30%)	245 (39%)	110 (26%)
• male	150 (71%)	68 (69%)	380 (60%)	312 (72%)
• unknown	0 (0%)	1 (1%)	4 (1%)	9 (2%)
Age [years]	46 [35 – 55]	41 [30 – 53]	45 [32 – 54]	41 [31 – 50]
Height [cm]	175 ± 8 [2]	176 ± 9 [0]	174 ± 10 [6]	176 ± 9 [8]
Weight [kg]	72 ± 11 [0]	72 ± 11 [0]	73 ± 13 [8]	71 ± 11 [1]
Body Mass Index	23.2 ± 2.6 [2]	23.2 ± 2.3 [0]	23.8 ± 3.3 [12]	22.8 ± 2.4 [9]
Country				
• Austria	4 (2%)	2 (2%)	15 (2%)	22 (5%)
• Benelux	12 (6%)	4 (4%)	20 (3%)	18 (4%)
• Switzerland	125 (59%)	41 (42%)	195 (31%)	48 (11%)
• Germany	36 (17%)	34 (35%)	85 (14%)	96 (22%)
• France	4 (2%)	2 (2%)	22 (3%)	36 (8%)
• Great Britain	6 (3%)	8 (8%)	88 (14%)	12 (3%)
• Italy	9 (4%)	4 (4%)	10 (2%)	125 (29%)
• United States	2 (1%)	0 (0%)	72 (11%)	3 (1%)
• Other	11 (5%)	1 (1%)	111 (18%)	59 (14%)
• Unknown	3 (1%)	2 (2%)	11 (2%)	12 (3%)
Sporting activities per week [h]	5.00 [2.50–8.00]	7.25 [5.00–13.50]	5.00 [2.00–10.00]	8.00 [4.75–14.00]

The mean age of the study population was 43 ± 13 years, height 175 ± 9 cm, body weight 72 ± 12 kg, and body mass index 23 ± 2.8. Most of the mountaineers were from Switzerland (30%), followed by Germany (18%), Italy (11%), and others as shown in Table 1. The participants engaged 9 ± 11 hours per week in sport activities. The language of the questionnaires had no effect on any of the assessed parameters.

About 20% of the population was taking regular medication that was not altitude-related, and 15% had taken medication against acute high-altitude illness during the last 48 hours (ibuprofen: 6.5%, acetaminophen: 4.3%, acetazolamide: 1.5%, diclofenac: 1.2%, ketoprofen or naproxen: 1.3%, and metoclopramide: 0.2%). Ninety-five percent of those who had taken acetazolamide ascended to the Margherita hut (4559 m), and 5% ascended to the Mönchsjoch hut (3650 m).

### 3.2 Prevalence of AMS

**Fig 1:**
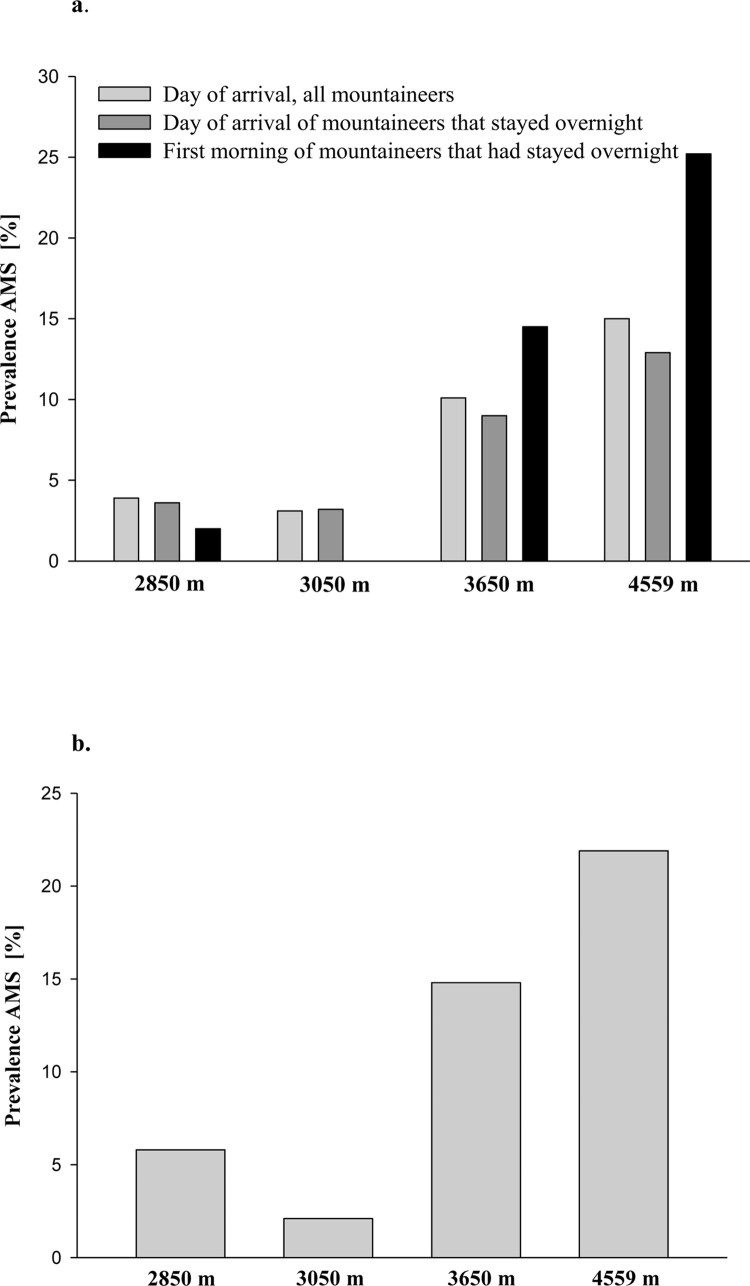
***a*)** Prevalence of AMS at the various altitudes according to the AMS-C score (i.e., > 0.7 points). In those who stayed overnight the AMS-C score was additionally assessed on the next morning. The numbers of mountaineers assessed at each time point are displayed in Table 1. ***b*)** Prevalence of AMS at the various altitudes according to the LLS (i.e., ≥ 3 points in presence of headache and at least one other symptom). The LLS was assessed only once, i.e., in all mountaineers on the day of arrival. 2850 m = Konkordia hut, 3050 m = Finsteraarhorn hut, 3650 m = Mönchsjoch hut, 4559 m = Margherita hut.

Fig 1 shows the prevalence of AMS at the four different altitudes determined by the AMS-C score (Fig 1A) and the LLS (Fig 1B). On the day of ascent, the prevalence of AMS according to the LLS and AMS-C score was 5.8 and 3.9% at 2850 m, 2.1 and 3.1% at 3050 m, 14.8 and 10.1% at 3650 m, and 21.9 and 15% at 4559 m. The prevalence of AMS by AMS-C scores were not different between the day of arrival and the next morning at 2850 m and 3050 m, and it increased from 10.1 to 14.5% and from 15 to 25.2% at 3650 m and 4559 m, respectively.

### 3.3 AMS severity

AMS scores were not different between 2850 m and 3050 m (Table 2) and did not increase overnight. They were higher at 3650 m and highest at 4559 m and the AMS-C score increased at these altitudes overnight by 0.07 and 0.14 points, respectively (Table 2). The distribution of these scores at all altitudes is shown in S1 and S2 Figs.

**Table 2:** Mountaineer’s AMS scores and risk factors at four different altitudes, data presented as mean ± standard deviation or count (%).

Characteristics	2850 m (Konkordia hut)	3050 m (Finsteraarhorn hut)	3650 m (Mönchsjoch hut)	4559 m (Margherita hut)
• Evening LLS	0.82 ± 1.04	0.78 ± 1.00	1.22 ± 1.33	1.63 ± 1.45
• Evening AMS-C score (all subjects)	0.14 ± 0.22	0.13 ± 0.23	0.26 ± 0.42	0.36 ± 0.47
• Evening AMS-C score of those staying overnight	0.13 ± 0.22	0.13 ± 0.23	0.26 ± 0.48	0.36 ± 0.48
• Morning AMS-C score	0.11 ± 0.25	0.09 ± 0.14	0.33 ± 0.50	0.50 ± 0.62
• AMS history score	3.23 ± 2.7	3.56 ± 2.49	5.06 ± 3.67	4.11 ± 2.88
• Subjects with AMS susceptibility	69 (32%)	42 (43%)	253 (40%)	188 (44%)
• Subjects without AMS susceptibility	103 (49%)	47 (48%)	150 (24%)	152 (35%)
• Subjects <1 day/year above 3000m	29 (14%)	1 (1%)	155 (25%)	29 (7%)
• unknown	11 (5%)	8 (8%)	72 (11%)	62 (14%)
• Days >3000 m in last 2 months	4.02 ± 8.96	5.21 ± 6.35	2.62 ± 5.57	7.75 ± 10.08
• Subjects with pre-acclimatization	39 (18%)	27 (28%)	74 (12%)	180 (42%)
• Subjects without pre-acclimatization	171 (81%)	68 (69%)	512 (81%)	211 (49%)
• Unknown	2 (1%)	3 (3%)	44 (7%)	40 (9%)
• Days of ascent	1.54 ± 1.04	2.36 ± 0.88	1.31 ± 0.81	3.29 ± 1.28
• Subjects with slow ascent	60 (28%)	90 (92%)	38 (6%)	176 (41%)
• Subjects with fast ascent	132 (62%)	7 (7%)	459 (73%)	236 (55%)
• Unknown	20 (9%)	1 (1%)	133 (21%)	19 (4%)
• Subjects with mountain guide	178 (84%)	70 (71%)	125 (20%)	287 (67%)
• Subjects without mountain guide	32 (15%)	28 (29%)	495 (79%)	133 (31%)
• Unknown	2 (1%)	0	9 (1%)	11 (2%)
• Altitude sickness knowledge score	3.5 [2.5–4.5]	4.5 [3.0–6.5]	3.5 [2.5–4.5]	3.5 [2.5–4.5]
• Unknown	2	0	1	1

### 3.4 Risk factors for AMS

**Fig 2:**
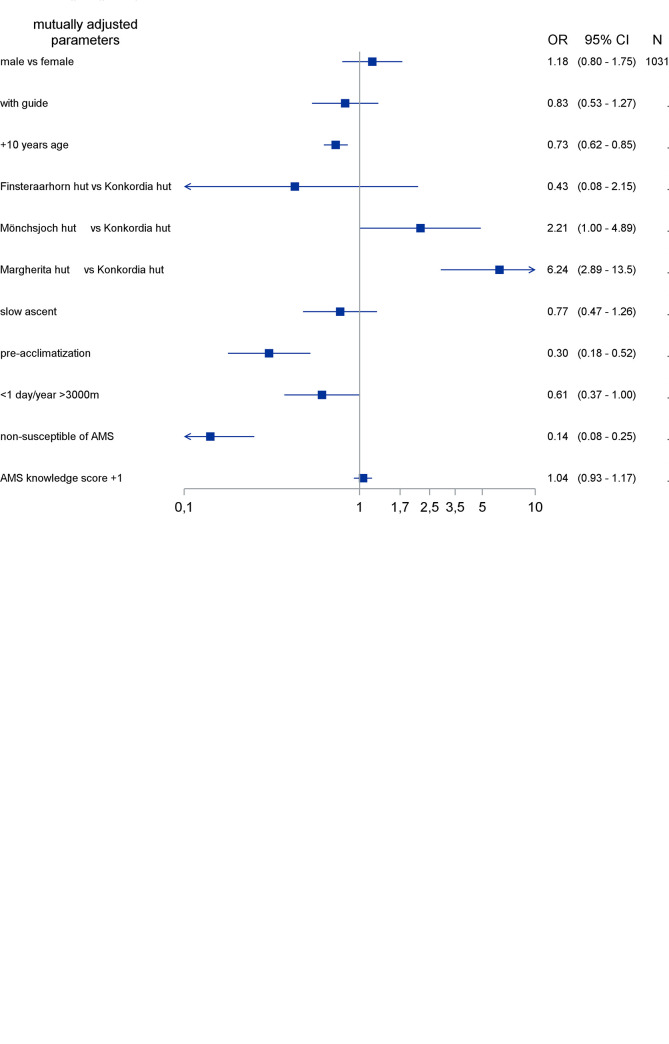
Analysis of risk factors for developing AMS based on the AMS-C score (2a) and the LLS (2b), respectively. The X-axis represents the odds ratio (OR); each row on the Y-axis represents an individual factor. The black boxes represent point estimates and corresponding 95% confidence intervals (CIs) of the individual factors. The effect of age was analyzed as age in years divided by 10 (1 unit change representing 10 years age difference).

Adjusted logistic regression models including age, history score, pre-acclimatization status, speed of ascent, leading by mountain guides, and altitude illness knowledge score were calculated for AMS defined by the AMS-C (Fig 2A) and by the LLS (Fig 2B). Both analyses show that an increase with higher altitude, a higher history score, a low degree of pre-acclimatization, and younger age were independent risk factors for developing AMS. Slower speed of ascent was consistently associated with reduced risk of AMS (all OR estimates below 0.8). Associations between AMS and the altitude illness knowledge score, sex, and being led by any type of mountain guides were weak and inconsistent. However, professional mountain guides vs. no guides showed a reduced odds ratio for developing AMS in the crude unadjusted analysis: 0.63 (CI: 0.41–0.95) with the AMS-C score, and 0.54 (CI: 0.37–0.77) with the LLS, respectively. Analyses of AMS-C values after the overnight stay confirm these findings (S3 Fig). Of note, including sleep disturbance into the LLS did not change the outcome of the multivariate analysis.

### 3.5 Altitude illness knowledge score

The mean overall knowledge score for the whole study population over all 5 questions was 3.61 ± 1.67 (minimum: 0, maximum: 10) and varied between 3.48 (Mönchsjoch hut) and 4.61 (Finsteraarhorn hut, see also Table 2). Mountaineers staying overnight had an average score of 3.95 (95% CI: 3.82–4.08), which was significantly higher than the average 3.20 (95% CI: 3.09–3.32) from day-tourists. S1 Table shows the scores obtained in each of the 5 questions.

### 3.6 Comparison of AMS symptoms with data obtained in 1990

As shown in Table 3, less mountaineers in the present study developed headache (OR: 0.72; CI: 0.57–0.92) and gastrointestinal upset (OR: 0.23; CI: 0.16–0.29) compared to about 30 years ago [6], while more mountaineers experienced dizziness (OR: 3.08; CI: 2.10–4.51). Overall, in the present study more mountaineers were free off any of the above-mentioned symptoms (OR: 0.53; CI: 0.41–0.69). The differences between the two studies increased with higher altitude.

**Table 3:** Comparison of AMS symptoms as reported in 1990 by Maggiorini et al. [6] and in the present study.

	2850 m (Konkordia hut)		3050 m (Finsteraarhorn hut)		3650 m (Mönchsjoch hut)		4559 m (Margherita hut)		Comparison symptom prevalence* OR (95% CI)
	1990 (n = 47)	2018 (n = 212)	1990 (n = 128)	2018 (n = 98)	1990 (n = 82)	2018 (n = 629)	1990 (n = 209)	2018 (n = 431)	2018 versus 1990 (n = 1370 versus 466)
Headache									
• *None (LLS 0)*	94%	76%	91%	80%	63%	64%	28%	52%	0.72 (0.57–0.92)
• *Any (LLS ≥ 1)*	6%	24%	9%	20%	37%	36%	72%	48%	
Gastrointestinal upset									
• *None (LLS 0)*	94%	94%	92%	94%	78%	95%	54%	88%	0.23 (0.16–0.29)
• *Any (LLS ≥ 1)*	6%	6%	8%	6%	22%	5%	46%	12%	
Dizziness									
• *No (LLS 0)*	100%	92%	99%	97%	92%	71%	87%	73%	3.08 (2.10–4.51)
• *Any (LLS ≥ 1)*	0%	8%	1%	3%	8%	29%	13%	27%	
Any symptoms									
• *No (LLS 0)*	53%	49%	41%	50%	26%	38%	8%	26%	0.53 (0.41–0.69)
• *Yes (LLS ≥ 1)*	47%	51%	59%	50%	74%	62%	92%	74%	

Comparable are entries concerning no symptoms. The remaining cumulation of any symptoms was derived as difference (set in italics). Prevalence of any symptoms were compared between the studies through logistic regression analyses, conditioning on huts as strata. Results are presented as odds ratio (OR) with 95% confidence intervals (95% CI).

*Comparison within huts as strata (conditional logistic regression)

## 4. Discussion

This study performed in four alpine huts located between 2850 and 4559 m shows that a history of AMS, higher altitude, a low degree of pre-acclimatization, younger age and speed of ascent to be clearly and consistently associated with risk for developing AMS, whereas associations for sex and pre-existing knowledge about AMS and HACE appeared indistinct. A higher proportion of individuals without headache in the present study suggests that the prevalence of AMS has most likely decreased at these huts compared to 1990 [6].

### 4.1 Prevalence and severity of AMS

The prevalence of AMS assessed by both scores was ≤ 6% at 2850 m and 3050 m and increased to 10–15% at 3650 m and 15–25% at 4559 m depending on the scoring system and the time of assessment. S2 Table shows a comparison of those who have with those who have no AMS according to the LLS and the AMS-C score at the same time and location. The prevalences of AMS found in this study are in accordance with investigations reporting an AMS prevalence of about 4–7% for altitudes between 2850 and 3050 m [8, 11, 12], of 11% at 3400 m [13], and of 28.4% at 4559 m [9]. The altitude difference of 200 m between the lowest 2 huts (i.e., the Konkordia and Finsteraarhorn hut) did not increase the prevalence of AMS, as others had observed [6, 8, 14].

Evening and morning scores and prevalence of AMS were not different at altitudes around 3000 m, which is most likely due to mild AMS at these locations. AMS prevalence increased over night by 5% and 10% at 3650 and 4559 m, respectively, which is in accordance with previous studies at comparable altitudes [15–17]. Our data underscore the conclusion of Beidleman et al. [14] that maximal AMS, at least at altitudes > 3000 m, should be assessed after exposures lasting about 20 hours.

A direct comparison of the lower prevalence of AMS obtained in the present study with those reported in 1990 [6] is not possible because different diagnostic criteria were applied. As shown in Table 3, more mountaineers had, however, no symptoms of AMS at all in the present vs the earlier study (OR: 0.53; CI: 0.41–0.69), and headache was also less frequent (OR: 0.72; CI: 0.57–0.92). Since headache is considered a compulsory symptom of AMS today, it follows that the prevalence of AMS in the present study was most likely lower compared to 30 years ago.

Because AMS is characterized by subjective, non-specific symptoms and there are no biomedical tests for diagnosing AMS, it cannot be excluded that other symptoms independent from altitude (e.g., gastrointestinal infections or migraine) might have affected the severity and prevalence of AMS assessed by the LLS and the AMS-C score, respectively.

### 4.2 Results on risk factors for developing AMS

Adjusted logistic regression models identify the same risk factors for AMS defined by either the AMS-C or the LLS, namely history of AMS, altitude, pre-acclimatization, and younger age.

The history of AMS was the strongest predictor. Those mountaineers classified as non-susceptible had a seven to nine times lower risk for AMS compared to those who frequently developed headache with additional symptoms above 3000 m. This finding is in agreement with studies performed in various settings of exposures [9, 18–21]. Further well established risk factors are confirmed in this study: pre-acclimatization (> 4 days above 3000 m in the last 2 months) [9] and higher altitude of the exposure [3]. A meta-analysis of Meier et al. [3] found that for each 1000 m increase in altitude above 2500 m, AMS prevalence increases by about 13%. Our data confirm this calculation with a prevalence of 4–6% at 2850 m and 3050 m, and of 25% at 4559 m. The reduction of AMS prevalence with pre-acclimatization (OR: 0.30; CI 0.18–0.52; and OR: 0.38; CI 0.20–0.71 for the LLS and the AMS-C score, respectively) matches well with the 2–3 fold reduction of AMS at 4559 m in a previous study [9].

We found that age was inversely related to AMS prevalence. Younger age as a risk factor has been identified before in other studies [21–28], but not in all [9, 29, 30]. In the present study, adults at an age between 18 to 30 years had the highest risk for AMS. For every further decade of life (up to > 60 years) AMS risk decreased. We can exclude that faster ascent or lower degree of pre-acclimatization account for the observed differences. However, behavioural and/or biological factors associated with aging could be responsible for the decrease in AMS risk with older age.

Crude analyses and distinguishing between professional and non-professional mountain guides consistently resulted in reduced OR estimates of AMS for professional guidance compared to no guides as shown in the result section. The weak and inconsistent association between AMS and sex in our sample including 446 (33%) women is in agreement with previous studies with large samples [6, 9, 31, 32], and smaller studies reporting more AMS in either women [33, 34] or in men [35, 36].

Slower speed of ascent is in our data associated with adjusted ORs between 0.77 (95% CI: 0.47–1.26) and 0.56 (95% CI: 0.31–1.00) for AMS according to the LLS (evening) and the AMS-C score from morning scores, respectively, which is less than reported in other studies [9]. The usual ascent to the Mönchsjoch hut occurs in one day from low altitude to the Jungfraujoch (3450 m) by train with a subsequent 1 hour walk to the hut at 3650 m. 73% ascended fast, 6% ascended slow (21% unknown) to this location (Table 2). Thus, it is conceivable that lack of variability of ascent rate in almost half of the study population prevented a clear association between mode of ascent and AMS. This hypothesis is supported by data obtained at the Margherita hut (4559 m), where the variability of the ascent rate is considerable (Table 2), and slow ascent appears to be protective (OR 0.67, CI 0.38–1.19) and by data of large prospective [21, 32], cross-sectional [9], and observational [9, 21, 31] studies reporting ascent rate as a major predictive factor.

### 4.3 Altitude illness knowledge score

While it appears plausible that well-informed mountaineers are less likely to suffer from acute high-altitude diseases [37], our study shows that greater knowledge was not associated with a lower risk for AMS. The overall knowledge about AMS and HACE assessed by questions addressing minimum knowledge for laypersons as recently defined by a group of experts in high-altitude medicine [10] was, however, unsatisfactory at all four altitudes (overall mean score 3.61 ± 1.67 out of a maximum of 12 points). Therefore, the power for finding a relationship between knowledge and AMS is low. Furthermore, we did not test the hypothesis of better knowledge leading to less AMS by an intervention improving knowledge.

The finding of little knowledge is in line with studies showing that trekkers and mountaineers in Nepal [22, 38], on Mount Kilimanjaro [39], and in Peru [40] had a low awareness regarding risk and prevention of AMS and were insufficiently prepared to recognize the symptoms and signs of AMS. Interestingly, 42% of the mountaineers in the present study achieved the maximum score regarding the most important risk factors for developing AMS, while the correct answers to other questions were ≤ 17% (see S1 Table). It is tempting to speculate that the better knowledge about risk factors has contributed to the lower prevalence of AMS in the present compared to the 1990 study [6] suggesting that improved knowledge about acute high altitude illnesses could be an important protective factor.

### 4.4 Limitations

We cannot exclude that the prevalence of AMS might have been underestimated somewhat due to mountaineers with severe AMS turning back before reaching the huts or refusing to participate due to malaise. However, underestimation can be expected to be low [9].

The evaluation of risk factors for AMS may have been affected by the relatively high percentage of day tourists (i.e., 46%) at the Mönchsjoch hut (3650 m) and the Margherita hut (4559 m). However, excluding day tourists from the adjusted logistic regression model did not change the identified risk factors. Because affiliation to mountaineering groups was not recorded, it is unclear how far potential clustering may have affected the results. The high percentage of day tourists may also lead to underestimation of AMS prevalence and severity, because in this population the time spent at the hut may have been too short to allow the development of full AMS symptomology.

Given the differences in accessibility of the four study locations it is also conceivable that differences in the population characteristics between the huts that were not assessed in the study may have affected the observed prevalence rates of AMS.

Furthermore, the lack of variability in ascent rate to the Mönchsjoch hut, which applies to 46% of the total sample likely explains the attenuated effect of ascent rate on AMS with ascent rate, as discussed in more detail above.

## 5. Conclusion

The present study shows that AMS is frequently experienced at altitudes of 3650 m and above. Compared to a study that was performed at the same locations about 30 years ago, less mountaineers developed altitude-related symptoms typical of AMS. The individual history of AMS, absolute altitude attained, extent of pre-acclimatization, ascent rate, and age were assessed as major risk factors for developing AMS. Greater knowledge about AMS and HACE was not directly associated with reduced risk.

## Supporting information

S1 Questionnaire Questionnaire evaluating the knowledge about acute high altitude illnesses. (DOCX)

S1 Table Altitude illness knowledge score. **Proportion of respondents receiving the maximum score and zero points per question.** The focus of each question was: Question 1: Typical symptoms of AMS. Question 2: Typical symptoms of HACE. Question 3: Lowest altitude at which AMS usually occurs. Question 4: Risk factors contributing to AMS and HACE. Question 5: Treatment of AMS and HACE. For details see S1 Questionnaire.
(DOCX)

S2 Table Comparison of those who have with those who have no AMS according to the LLS and the AMS-C score at the same time and location. Overall agreement of the two scores is good with concordance (= percent of equally categorized subjects) of 91%. However, the differences in case specification suggest that the two definitions may capture somewhat different disease entities.
(DOCX)

S1 Fig Analysis of risk factors for developing AMS based on the AMS-C morning scores. The X-axis represents the odds ratio (OR); each row on the Y-axis represents an individual factor. The black boxes represent point estimates and corresponding 95% confidence intervals (CIs) of the individual factors. The effect of age was analyzed as age in years divided by 10 (1 unit change representing 10 years age difference).
(DOCX)

S2 Fig Distribution of the AMS-C score. In the evening (4a) and the next morning (4b) at the Konkordia hut (2850 m), Finsteraarhorn hut (3050 m), Mönchsjoch hut (3650 m), and Margherita hut (4559 m).
(DOCX)

S3 Fig Distribution of the Lake Louise score. In the evening (3) at the Konkordia hut (2850 m), Finsteraarhorn hut (3050 m), Mönchsjoch hut (3650 m), and Margherita hut (4559 m).
(DOCX)

S1 File (RTF)

S2 File (CSV)
