# Supplementary material for: Prevalence and knowledge about acute mountain sickness in the Western Alps
Source: PLoS One. 2023 Sep 14;18(9):e0291060. doi: 10.1371/journal.pone.0291060 (PMC10501682; doi:10.1371/journal.pone.0291060)
Supplement: S1 Questionnaire — (DOCX) [file pone.0291060.s001.docx]

**Supplement 1**

**1. The altitude illness knowledge score was assessed with the following questions:**

(Each question-score was limited to a minimum of zero points, missed out correct answers were not penalized. In summary over all 5 questions a maximum score of 12 points could be achieved).

***1. Which of the following are typical symptoms of acute mountain sickness?***

| - Nausea / loss of appetite | - Nosebleed |
| --- | --- |
| - Ataxia / dysbalance | - Shortness of breath |
| - Breathing-dependent pain | - Tingling in fingers |
| - Abdominal pain / cramps | - Loss of consciousness |
| - Fever | - Diarrhea |
| - Dizziness | - Cough |
| - Deemed vision | - Confusion |
| - Headache |  |

*Correct answers required selection of headache plus nausea / loss of appetite, and dizziness. Each correct answer was awarded with 1 point, with 1 point penalty withdrawn for each incorrect response (maximum score 3).*

***2. Which of the following are typical symptoms of high-altitude cerebral edema?***

| - Nausea / loss of appetite | - Nosebleed |
| --- | --- |
| - Ataxia / dysbalance | - Shortness of breath |
| - Breathing-dependent pain | - Tingling in fingers |
| - Abdominal pain / cramps | - Loss of consciousness |
| - Fever | - Diarrhea |
| - Dizziness | - Cough |
| - Deemed vision | - Confusion |
| - Headache |  |

*Maximum score required selection of ataxia, fever, dizziness, headache, confusion, and loss of consciousness, each awarded with 0.5 points, incorrect answers penalized with 0.5 (maximum score 3).*

***3. What is the lowest altitude at which acute mountain sickness may develop?***

a) < 1500 m b) 1500 – 2500 m c) 2600 – 3500 m d) 3600 – 4500 m e) 4600 – 5000 m f) > 5000 m

*For the correct answer (1500 – 2500 m) 1 point was given.*

***4. Which of the following are the two most important risk factors for developing acute mountain sickness?***

| - High age | - Gaining altitude too quickly | - Poor nutrition |
| --- | --- | --- |
| - Exercise | - Lack of acclimatization | - Dehydration |
| - Female gender | - Male gender | - Poor physical fitness |

*Maximum score required selection of gaining altitude too quickly, and lack of acclimatization (1 point per answer as award or penalty, maximum score 2).*

***5. Which of the following statements is / are correct?***

a) High-altitude cerebral edema is usually preceded by acute mountain sickness.

b) In case of high-altitude cerebral edema it is advised to descent immediately, and (if possible) to give oxygen and dexamethasone (cortisone)

c) High-altitude cerebral edema is usually harmless and does not require therapy.

d) Incipient acute mountain sickness may be treated with a day of rest and painkillers.

*For a maximum score the respondent had to know that high-altitude cerebral edema is usually preceded by AMS, that in case of high-altitude cerebral edema it is advised to descent immediately, and – if possible – to give oxygen and dexamethasone (cortisone), and that an incipient AMS may be treated with a day of rest and painkillers. Maximum score 3.*
