# Supplementary material for: Prevalence and knowledge about acute mountain sickness in the Western Alps
Source: PLoS One. 2023 Sep 14;18(9):e0291060. doi: 10.1371/journal.pone.0291060 (PMC10501682; doi:10.1371/journal.pone.0291060)
Supplement: S1 Table — Proportion of respondents receiving the maximum score and zero points per question. The focus of each question was: Question 1: Typical symptoms of AMS. Question 2: Typical symptoms of HACE. Question 3: Lowest altitude at which AMS usually occurs. Question 4: Risk factors contributing to AMS and HACE. Question 5: Treatment of AMS and HACE. For details see S1 Questionnaire. (DOCX) [file pone.0291060.s002.docx]

**Supplement 2**

Altitude illness knowledge score. Proportion of respondents receiving the maximum score and zero points per question. The focus of each question was: Question 1: Typical symptoms of AMS. Question 2: Typical symptoms of HACE. Question 3: Lowest altitude at which AMS usually occurs. Question 4: Risk factors contributing to AMS and HACE. Question 5: Treatment of AMS and HACE. For details see Supplement 1.

|  | **2850 m**  **(Konkordia hut)** | **3050 m**  **(Finsteraarhorn hut)** | **3650 m**  **(Mönchsjoch hut)** | **4559 m**  **(Margherita hut)** | **All altitudes and huts combined** |
| --- | --- | --- | --- | --- | --- |
| **Question 1**  - 0 points  - max. (3) points  - mean score | 76 %  2 %  0.37 ± 0.70 | 65 %  4 %  0.61 ± 0.93 | 86 %  1 %  0.24 ± 0.60 | 76 %  1 %  0.27 ± 0.61 | 80 %  1 %  0.32 ± 0.67 |
| **Question 2**  - 0 points  - max. (3) points  - mean score | 40 %  0 %  0.65 ± 0.63 | 29 %  0 %  0.87 ± 0.73 | 39 %  0 %  0.64 ± 0.65 | 45 %  0 %  0.63 ± 0.67 | 40 %  0 %  0.66 ± 0.67 |
| **Question 3**  - 0 points  - max. (1) point  - mean score | 78 %  22 %  0.21 ± 0.41 | 68 %  32 %  0.32 ± 0.47 | 83 %  17 %  0.20 ± 0.40 | 88 %  12 %  0.16 ± 0.37 | 83 %  17 %  0.19 ± 0.39 |
| **Question 4**  - 0 points  - max. (2) points  - mean score | 16 %  42 %  1.26 ± 0.71 | 10 %  55 %  1.45 ± 0.68 | 22 %  36 %  1.14 ± 0.74 | 14 %  49 %  1.18 ± 0.74 | 18 %  42 %  1.24 ± 0.73 |
| **Question 5**  - 0 points  - max. (3) points  - mean score | 30 %  14 %  1.04 ± 0.85 | 18 %  10 %  1.37 ± 0.90 | 21 %  8 %  1.24 ± 0.86 | 26 %  6 %  1.16 ± 0.86 | 24 %  8 %  1.19 ± 0.87x |
