# Supplementary material for: Prevalence and knowledge about acute mountain sickness in the Western Alps
Source: PLoS One. 2023 Sep 14;18(9):e0291060. doi: 10.1371/journal.pone.0291060 (PMC10501682; doi:10.1371/journal.pone.0291060)
Supplement: S2 Table — Overall agreement of the two scores is good with concordance (= percent of equally categorized subjects) of 91%. However, the differences in case specification suggest that the two definitions may capture somewhat different disease entities. (DOCX) [file pone.0291060.s003.docx]

**Supplement 6**

Comparison of those who have with those who have no AMS according to the LLS and the AMS-C score at the same time and location.

|  | No AMS according to LLS | AMS according to LLS | LLS missing or incomplete |
| --- | --- | --- | --- |
| No AMS: AMS-C <0.7 | 1070 | 85 | 28 |
| AMS with AMS-C ≥0.7 | 36 | 90 | 8 |
| AMS-C missing or incomplete | 25 | 18 | 10 |

Overall agreement of the two scores is good with concordance (= percent of equally categorized subjects) of 91 %. However, the differences in case specification suggest that the two definitions may capture somewhat different disease entities.
