# Supplementary material for: Prevalence and knowledge about acute mountain sickness in the Western Alps
Source: PLoS One. 2023 Sep 14;18(9):e0291060. doi: 10.1371/journal.pone.0291060 (PMC10501682; doi:10.1371/journal.pone.0291060)

**Supplement 3**

Analysis of risk factors for developing AMS based on the AMS-C morning scores. The X-axis represents the odds ratio (OR); each row on the Y-axis represents an individual factor. The black boxes represent point estimates and corresponding 95% confidence intervals (CIs) of the individual factors. The effect of age was analyzed as age in years divided by 10 (1 unit change representing 10 years age difference).


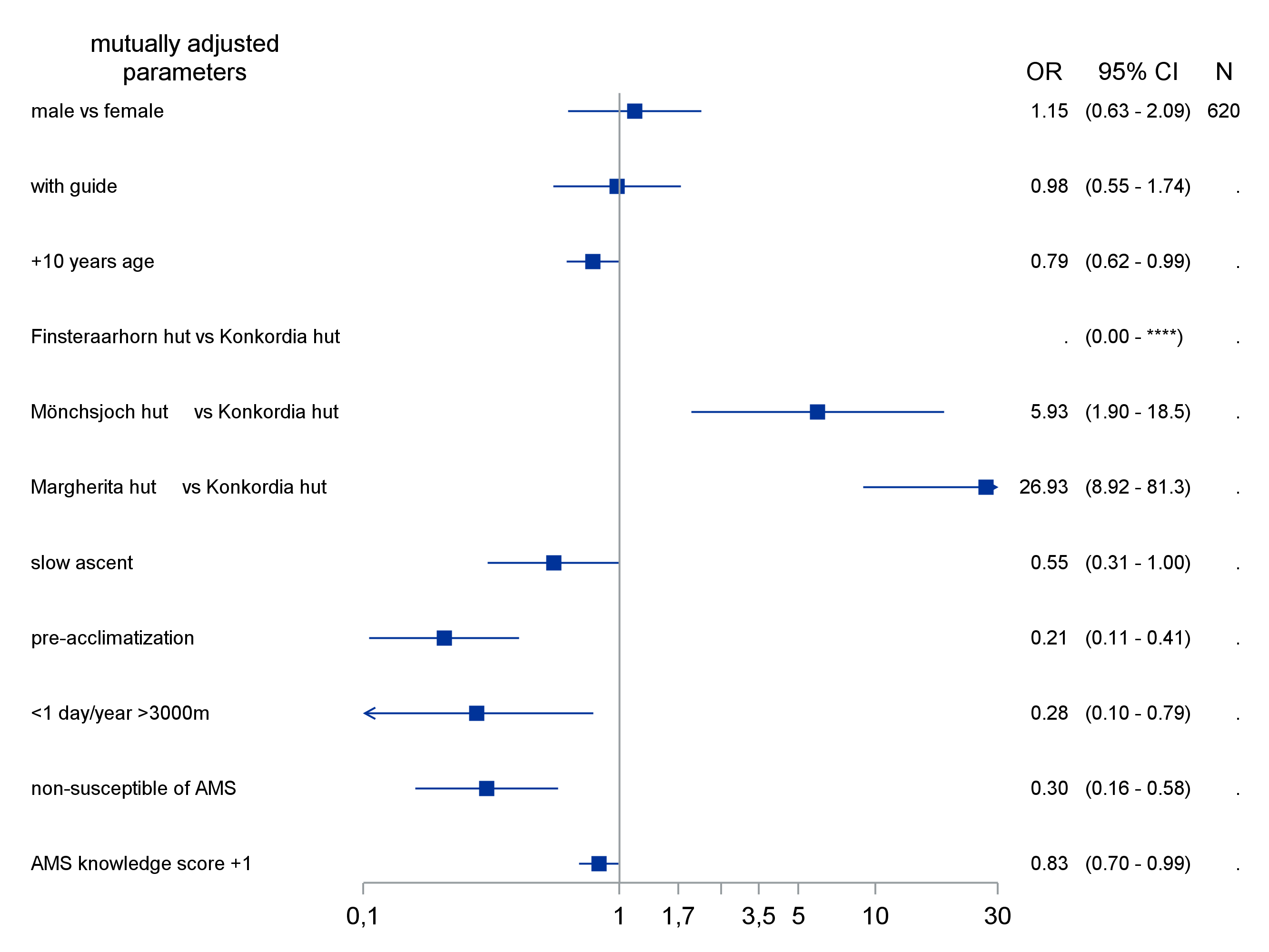

Supplement: S1 Fig — The X-axis represents the odds ratio (OR); each row on the Y-axis represents an individual factor. The black boxes represent point estimates and corresponding 95% confidence intervals (CIs) of the individual factors. The effect of age was analyzed as age in years divided by 10 (1 unit change representing 10 years age difference). (DOCX) [file pone.0291060.s004.docx]
