# Supplementary material for: Prevalence and knowledge about acute mountain sickness in the Western Alps
Source: PLoS One. 2023 Sep 14;18(9):e0291060. doi: 10.1371/journal.pone.0291060 (PMC10501682; doi:10.1371/journal.pone.0291060)

**Supplement 4**

Distribution of the AMS-C score in the evening (**4a**) and the next morning (**4b**) at the Konkordia hut (2850 m), Finsteraarhorn hut (3050 m), Mönchsjoch hut (3650 m), and Margherita hut (4559 m).

**4a)**


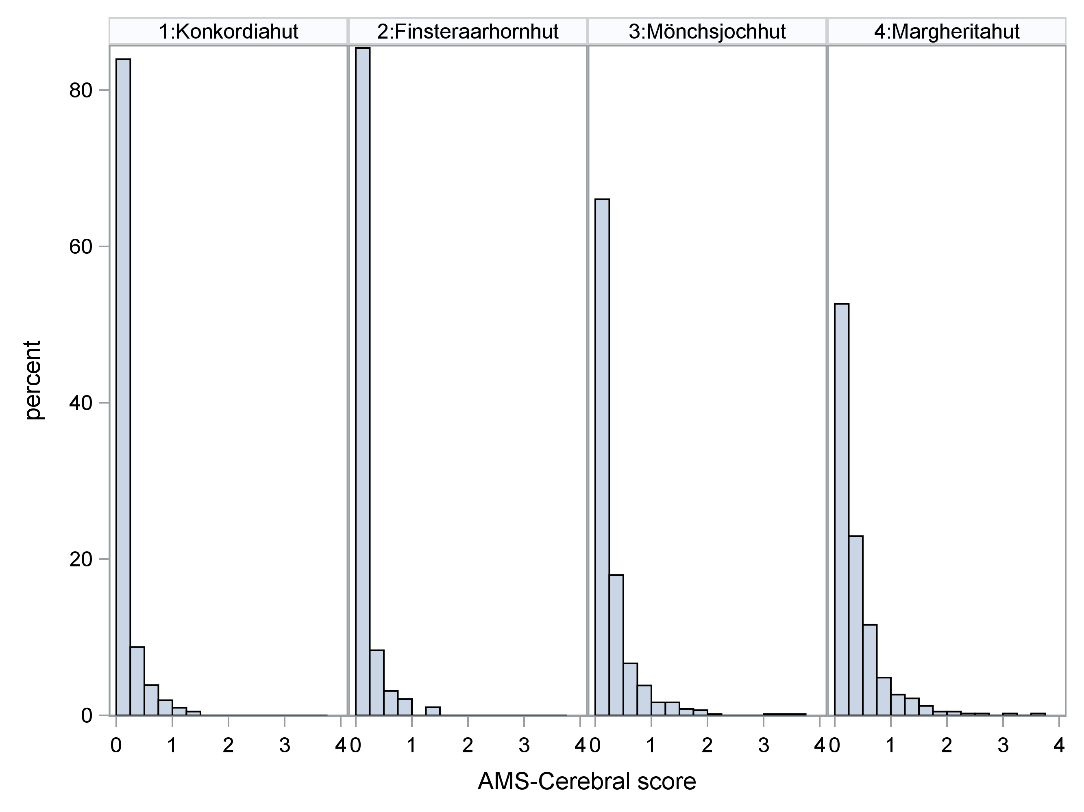


**4b)**


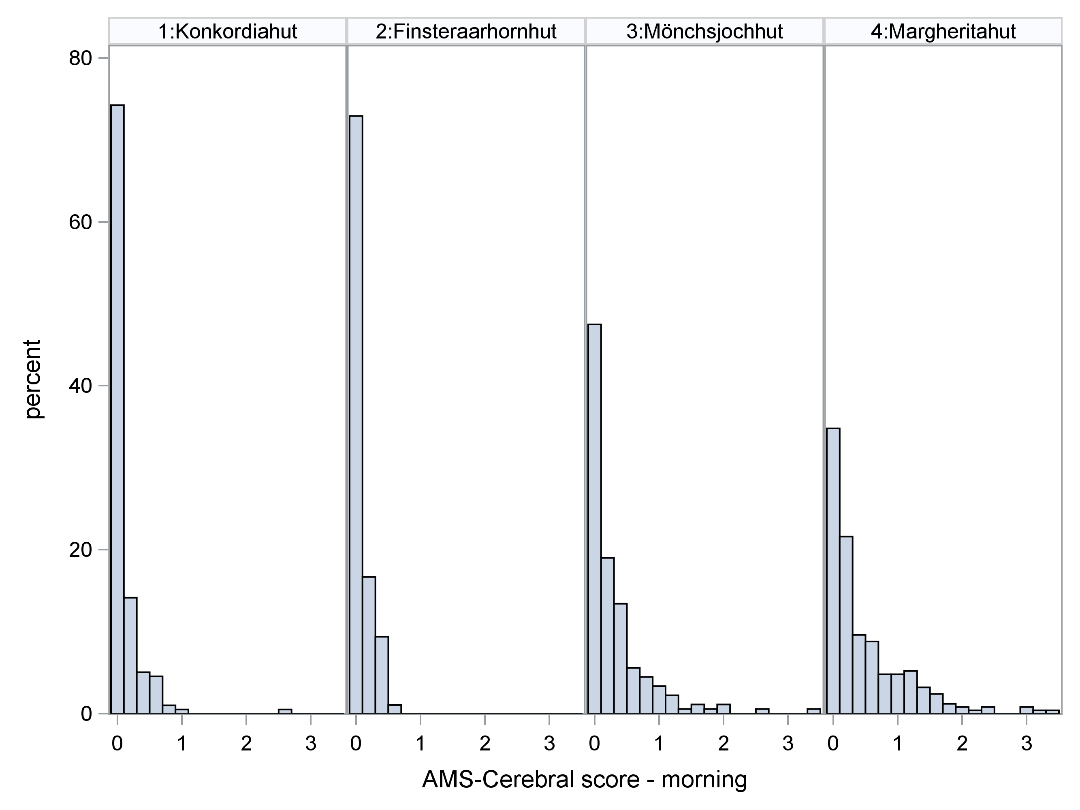

Supplement: S2 Fig — In the evening (4a) and the next morning (4b) at the Konkordia hut (2850 m), Finsteraarhorn hut (3050 m), Mönchsjoch hut (3650 m), and Margherita hut (4559 m). (DOCX) [file pone.0291060.s005.docx]
