# Supplementary material for: Prevalence and knowledge about acute mountain sickness in the Western Alps
Source: PLoS One. 2023 Sep 14;18(9):e0291060. doi: 10.1371/journal.pone.0291060 (PMC10501682; doi:10.1371/journal.pone.0291060)

**Supplement 5**

Distribution of the Lake Louise Score score in the evening (**3**) at the Konkordia hut (2850 m), Finsteraarhorn hut (3050 m), Mönchsjoch hut (3650 m), and Margherita hut (4559 m).


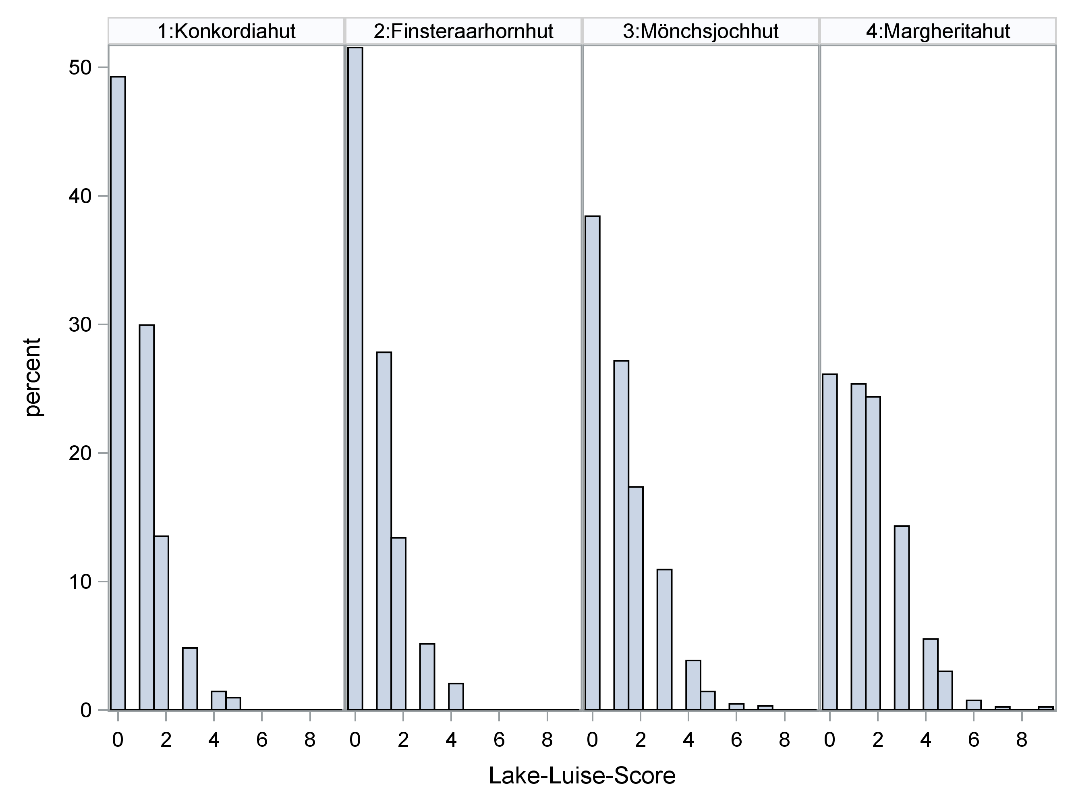

Supplement: S3 Fig — In the evening (3) at the Konkordia hut (2850 m), Finsteraarhorn hut (3050 m), Mönchsjoch hut (3650 m), and Margherita hut (4559 m). (DOCX) [file pone.0291060.s006.docx]
