# Supplementary material for: Prevalence and knowledge about acute mountain sickness in the Western Alps
Source: PLoS One. 2023 Sep 14;18(9):e0291060. doi: 10.1371/journal.pone.0291060 (PMC10501682; doi:10.1371/journal.pone.0291060)
Supplement: S1 File — (RTF) [file pone.0291060.s007.rtf]

Variables in Creation Order	
#	Variable	Type	Len	Format	Informat	Label	
1	ID	Char	10	$10.	$10.	ID	
2	Ort	Char	19	$19.	$19.	hut	
3	state	Char	3	$3.	$3.	nationality	
4	quest_q1	Num	8			Typical symptoms of AMS[score]	
5	quest_q2	Num	8			Typical symptoms for onset of high-altitude cerebral edema [score]	
6	quest_q3	Num	8			lowest height for AMS	
7	quest_q4	Num	8			risk factors for AMS (2 correct)	
8	quest_q5	Num	8			Mult-choice on AMS and high-alt. cerebral edema	
9	guide	Num	8			guided tour: 2=professional/1=experienced,qualified/0=none	
10	gender	Num	8			Sex 1=female/0=male	
11	age	Num	8			age	
12	amsc_1_behave	Num	8			AMSC - lightheaded, faint 0=no - 5=very strong	
13	amsc_2_headache	Num	8			AMSC - headache 0=no - 5=very strong	
14	amsc_3_dizzy	Num	8			AMSC - Dizziness 0=no - 5=very strong	
15	amsc_4_black	Num	8			AMSC - blackout 0=no - 5=very strong	
16	amsc_5_vell	Num	8			AMSC - dim vision 0=no - 5=very strong	
17	amsc_6_uncertain	Num	8			AMSC - uncertain coordination 0=no - 5=very strong	
18	amsc_7_weak	Num	8			AMSC - feeling weak 0=no - 5=very strong	
19	amsc_8_nausea	Num	8			AMSC - nausea 0=no - 5=very strong	
20	amsc_9_loss_app	Num	8			AMSC - loss of appetite 0=no - 5=very strong	
21	amsc_10_sick	Num	8			AMSC - sick 0=no - 5=very strong	
22	amsc_11_hungover	Num	8			AMSC - hungover 0=no - 5=very strong	
23	M_amsc_1_behave	Num	8			morning AMSC - lightheaded, faint 0=no - 5=very strong	
24	M_amsc_2_headache	Num	8			morning AMSC - headache 0=no - 5=very strong	
25	M_amsc_3_dizzy	Num	8			morning AMSC - dizziness 0=no - 5=very strong	
26	M_amsc_4_black	Num	8			morning AMSC - blackout 0=no - 5=very strong	
27	M_amsc_5_vell	Num	8			morning AMSC - dim vision 0=no - 5=very strong	
28	M_amsc_6_uncertain	Num	8			morning AMSC - uncertain coordination 0=no - 5=very strong	
29	M_amsc_7_weak	Num	8			morning AMSC - feeling weak 0=no - 5=very strong	
30	M_amsc_8_nausea	Num	8			morning AMSC - nausea 0=no - 5=very strong	
31	M_amsc_9_loss_app	Num	8			morning AMSC - loss of appetite 0=no - 5=very strong	
32	M_amsc_10_sick	Num	8			morning AMSC - sick 0=no - 5=very strong	
33	M_amsc_11_hungover	Num	8			morning AMSC - hungover 0=no - 5=very strong	
34	sprache	Char	10			Questionnaire language: D=German, F=French, E=English, I=Italian	
35	amsc_ge07	Num	8			Acute mountain sickness (AMSC >=0.7) 1=yes/0=no	
36	M_amsc_ge07	Num	8			morning Acute mountain sickness (AMSC >=0.7) 1=yes/0=no	
37	langsam	Num	8			Slow ascent 1=yes/0=no	
38	llq_headache	Num	8			LLQ: headache 0=no - 3=incapacitatingly	
39	llq_nausea	Num	8			LLQ: nausea 0=no - 3=incapacitatingly	
40	llq_tired	Num	8			LLQ: tiredness 0=no - 3=incapacitatingly	
41	llq_dizzy	Num	8			LLQ: dizziness 0=no - 3=incapacitatingly	
42	llq_sleepless	Num	8			LLQ:sleeplessness 0=no - 3=incapacitatingly	
43	AMS_rawllq3	Num	8			Acute mountain sickness (llq>=3 &headache) 1=yes/0=no	
44	vorakklim	Num	8			>= 5 days above 3000m over NN in last 2 months  1=yes/0=no	
45	hoehenanam	Num	8			history of AMS: 0=not assessible,1=no history, 2=potential history of AMS	
46	Fragscore5	Num	8			Sum of Quest.s 1-5, higher is better, 0-11.5 pts	
